# The association of screen time with childhood obesity and metabolic status: a mediation analysis of cardiorespiratory fitness

**DOI:** 10.3389/fendo.2025.1719372

**Published:** 2026-01-13

**Authors:** Jie Zhang, Bi-Lian Wang, Han Jin, Jia-Ying Gu, Jia-Hao Zhang, Guan-Fu Shou, Hui Wang, Ping-Ping Zhang, Li Li

**Affiliations:** 1 Department of Endocrinology and Metabolism, The First Affiliated Hospital of Ningbo University, Ningbo, Zhejiang, China; 2 Ningbo University Health Science Center, Ningbo, Zhejiang, China; 3 Ningbo Art Experimental School, Ningbo, Zhejiang, China; 4 Department of Endocrinology, The Second Hospital of Jiaxing, Jiaxing, Zhejiang, China; 5 Hangzhou Qingwu Health Management Limited Company, Hangzhou, Zhejiang, China; 6 Department of Maternal and Child Health, School of Public Health, Peking University, Beijing, China; 7 Ningbo Center for Healthy Lifestyle Research, Chronic Disease Management Office, The First Affiliated Hospital of Ningbo University, Ningbo, Zhejiang, China

**Keywords:** cardiorespiratory fitness, children, mediation effect, obesity, screen time

## Abstract

**Background:**

Childhood obesity and metabolic disorders are increasingly drawing global attention due to their long-term associations with chronic metabolic diseases. Excessive screen time is a key contributor to obesogenic behavior and is associated with unfavorable metabolic outcomes. This study aimed to analyze the relationships between screen time and obesity-related metabolic indicators in children and to explore the mediating role of cardiorespiratory fitness (CRF).

**Methods:**

Baseline data were drawn from the OptiChild study, involving 1,286 third-grade students in Ningbo, China. Anthropometric measurements, body composition, blood pressure, and fasting blood samples were collected. CRF was assessed using the 20-meter shuttle run test. Screen time, physical activity, and diet quality were assessed through questionnaires. Generalized linear mixed models were employed to analyze the associations, and mediation analysis was performed using bootstrap resampling.

**Results:**

After adjusting for sex, age, maternal education level, physical activity and diet, higher screen time was significantly associated with increased visceral fat area (VFA) (β = 0.029, P = 0.009), body fat mass index (BFMI) (β = 0.109, P = 0.017), body fat percentage (BFP) (β = 0.469, P = 0.010), but decreased high-density lipoprotein cholesterol (HDL-C) (β = -0.014, P = 0.038). Mediation analysis indicated that CRF explained 66.6% of the association between screen time and VFA (P<0.05), 67.5% with BFMI (P = 0.014), 65.1% with BFP (P = 0.006), and 22.6% with HDL-C (P = 0.026).

**Conclusion:**

In Chinese children, lower screen time was associated with more favorable obesity-related profiles, with CRF playing as a significant mediator in this association.

## 1 Introduction

The global prevalence of obesity among children and adolescents has risen dramatically over recent decades, posing significant public health challenges (1). A systematic review and meta-analysis covering 2000–2023 reported a global obesity prevalence of 8.5% (95% CI: 8.2–8.8) and overweight prevalence of 14.8% (95% CI: 14.5–15.1) among children and adolescents (2). The prevalence of obesity among children and adolescents varies significantly across different countries and regions, ranging from 0.4% in Vanuatu to 28.4% in Puerto Rico, with higher rates observed in high-income countries or regions (2). In China, data from the National Student Physical Fitness and Health Survey indicate that the obesity prevalence among children and adolescents aged 7 to 18 rose from 0.1% in 1985 to 9.6% in 2019 (3). The prevalence of overweight and obesity among urban children and adolescents in China is higher than that in rural areas; however, the gap between urban and rural regions is gradually narrowing (4). Excessive fat accumulation is mutually causative with abnormalities in various metabolic indicators (5, 6), and contributes to various chronic diseases such as cardiovascular diseases, dyslipidemia, and type 2 diabetes later in life (7–9).

Previous research indicated that sedentary behavior is a key factor in preventing unhealthy weight gain (10). Sedentary behavior is defined as sitting or lying down while awake, with an energy expenditure of ≤1.5 metabolic equivalents (METs) (11). Screen time refers to the duration spent using screen devices such as tablets, computers, or smartphones while sitting, standing, or engaging in light physical activity, and it is the most common sedentary behaviors. According to the International 24-Hour Movement Guidelines, children and adolescents aged 5–17 years are recommended to limit recreational screen time to no more than 2 hours per day (12, 13). However, data show that 45% to 80% of children and adolescents fail to meet this standard in Australia (13). Recent epidemiological data indicate that more than 70% of Chinese school-aged children exceed the recommended ≤2 hours/day of recreational screen time (3), a proportion comparable to or even higher than that observed in Australia (13), the United States (14), and South Korea (15). Excessive screen time is linked to various obesogenic behaviors, including increased sedentary time, physical inactivity, unhealthy dietary habits, and reduced sleep duration (16, 17).

Increasing evidence suggests that the use of electronic devices has a negative impact on childhood obesity. Research has identified a significant dose-response relationship between the daily time children spend watching television and the prevalence of overweight, while physical activity unable to effectively mitigate this risk (18, 19). Longitudinal cohort studies further demonstrate that increased television viewing during childhood predicts overweight and obesity in adulthood (20). In adults, being overweight and having poor health have been associated with watching television for more than 2 hours per day during childhood (21). Observational studies have also shown that prolonged screen time is linked to various metabolic risk factors, including hypertension, elevated cholesterol levels, insulin resistance, type 2 diabetes, and metabolic syndrome (22). Additionally, several experimental studies manipulating screen media exposure in children have demonstrated a causal relationship between screen media exposure and weight gain (23).

Cardiorespiratory fitness (CRF) refers to the ability of the circulatory and respiratory systems to deliver oxygen to skeletal muscle mitochondria to produce the energy required for physical activity (24). CRF is associated with various diseases, including cardiovascular disease, type 2 diabetes, and other metabolic risks (25, 26). It plays a crucial role in managing different metabolic risk factors such as blood glucose, lipid levels, blood pressure, and insulin sensitivity (27). Low CRF can lead to decreased metabolic capacity, thereby contributing to weight gain and the development of obesity. Excessive screen time is often linked to reduced physical activity, and a sedentary lifestyle negatively impacts heart and lung health, further leading to decreased energy expenditure. Although previous studies have identified independent associations between screen time, cardiorespiratory fitness (CRF), and obesity (28, 29), there is a lack of mediating studies on screen time and obesity among Asian populations. Furthermore, little is known about whether CRF mediates the impact of screen time on obesity-related metabolic indicators.

This study has two objectives. First, we aim to investigate the association between screen time and obesity-related metabolic health indicators. Second, we assess the mediating role of CRF in the relationship between screen time and obesity-related metabolic indicators.

## 2 Methods

### 2.1 Data source and participants

**Figure 1:**
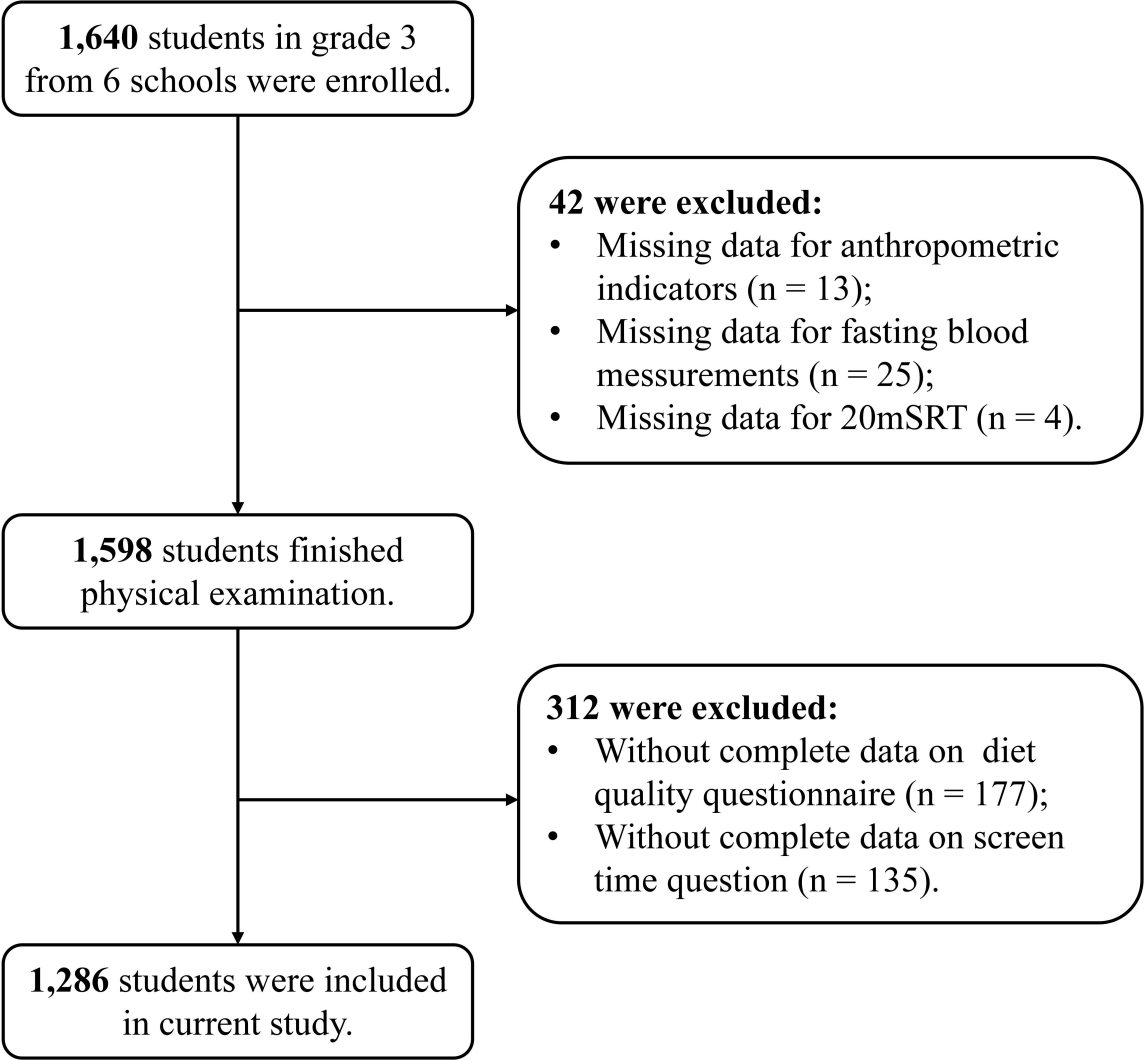
Flow chart for included participants.

This study is part of “Optimizing Intervention Effects in Children and Adolescents in Ningbo (OptiChild study)” program, designed as a cluster randomized controlled trial (30). The project team conducted a healthy lifestyle intervention study among third-grade students in six schools in Ningbo in September 2022, enrolling a total of 1,640 students. To investigate the associations between screen time and obesity and metabolic-related indicators, 1,286 participants were ultimately included in the study after excluding those with missing data, the enrollment process of the research subjects is illustrated in **Figure 1**. The project received ethical approval from the Ethics Committee of the First Affiliated Hospital of Ningbo University (Approval No. 2021-R168). All participating students and their primary guardians provided written informed consent.

### 2.2 Anthropometry

Children’s waist circumference (WC) and hip circumference (HC) were measured with a waist measurement tape, accurate to 0.1 centimeters. Height was measured with a mechanical stadiometer, also accurate to 0.1 centimeters. The waist-to-height ratio (WHtR) was calculated from the final waist circumference and height values, using the formula WHtR = WC (cm) / height (cm). The waist-to-hip ratio (WHR) was calculated from the final waist and hip circumference, using the formula WHR = WC (cm) / HC (cm).

Blood pressure was measured with an Omron digital blood pressure monitor (HEM-7121, Omron Corp, Kyoto, Japan), recording systolic blood pressure (SBP) and diastolic blood pressure (DBP). The measurement was repeated twice. If the difference between the two readings exceeded 10 mmHg, a third measurement was taken. Two readings within the acceptable error range were entered, and the average of these two measurements was used for data analysis. Mean arterial pressure (MAP) was calculated from the systolic and diastolic blood pressure values, using the formula MAP (mmHg) = 1/3 SBP (mmHg) + 2/3 DBP (mmHg).

Body composition was measured with a bioelectrical impedance analysis system (InBody770, InBody Co., Cerritos, California, USA), accurate to 0.1 kilograms. Participants were required to fast for at least eight hours before measurement. Measurement outcomes included weight, visceral fat area (VFA), body fat percentage (BFP), body fat mass (BFM), skeletal muscle mass (SMM), fat-free mass (FFM). Body mass index (BMI) was calculated from height and weight using the formula BMI (kg/m²) = weight (kg) / height (m)². Similarly, body fat mass index (BFMI), skeletal muscle mass index (SMI), and fat-free mass index (FFMI) were derived by dividing BFM, SMM, and FFM (all in kg) by height squared (m²), respectively.

### 2.3 Biochemical examinations

Blood samples were collected concurrently with the routine health examinations mandated by national guidelines. The participants were required to fast for at least 8 hours before sample collection, which was conducted by trained staff from local community health centers at the schools. Laboratory professionals from the First Affiliated Hospital of Ningbo University measured fasting plasma glucose (FPG), fasting insulin (FINS), triglycerides (TG), total cholesterol (TC), high-density lipoprotein cholesterol (HDL-c), and low-density lipoprotein cholesterol (LDL-c) using the remaining blood samples from routine tests. The samples were transported at 4 degrees Celsius and analyzed within 4 hours of collection. FINS was measured using a fully automated immunoassay analyzer (Roche Cobas E602, Basel, Switzerland) via chemiluminescence. FPG, TG, TC, LDL-c, and HDL-c were measured using enzyme methods on a fully automated analyzer (Beckman AU5800, California, USA). The homeostasis model assessment of insulin resistance (Homa-IR) index was calculated from FPG and FINS using the formula: Homa-IR = FINS (mU/L) × FBG (mmol/L) / 22.5 (31).

Cardiovascular metabolic risk scores (CMRs) are a useful indicator for assessing cardiovascular metabolic risk in children. Based on previous evidence, MAP, Homa-IR, TC/HDL-c, and TG were selected as components of CMRs in this study (32). For each component, z-scores were calculated by subtracting the sex- and age-specific mean and dividing by the corresponding standard deviation. The overall CMRs was computed as the sum of these z-scores using the formula: CMRs = Z (MAP) + Z (Homa-IR) + Z (TC/HDL-c) + Z (TG). A higher CMRs value indicates an increased cardiovascular metabolic risk for the individual.

### 2.4 Questionnaire survey

Demographic information, including date of birth, sex, and maternal education level, was collected by a parent questionnaire. Children's screen time was assessed using a self-designed questionnaire, which has been validated in prior studies and demonstrates good reliability and validity. The survey examined the average daily screen time during school days and weekends over the past seven days, covering the time spent using electronic devices such as watching television and using computers or smartphones (33). Screen time was categorized into two groups: ≤ 2 hours per day and >2 hours per day (34). The time spent in moderate or vigorous-intensity physical activity (MVPA) over the previous week was assessed using a self-reported Physical Activity Questionnaire (PAQ), which has been validated in Chinese children (35). Detailed information about the questionnaire has been described elsewhere. In this study, daily MVPA time was categorized as ≥ 60 minutes or < 60 minutes per day according to the recommendations of the World Health Organization (36).

The DQQ is a rapid assessment tool designed to evaluate dietary quality within populations. It can be used to construct various dietary quality indicators at the population level, including food group diversity scores and Global Dietary Recommendations (GDR) score. The GDR score serves to reflect dietary risk factors for non-communicable diseases, deriving the GDR-healthy score, GDR-limit score, and overall GDR score from dietary intake data. The overall GDR score is calculated using the formula: GDR total score = GDR-healthy - GDR-limit + 9, which reflects all 11 global dietary component recommendations. The range of the GDR total score is from 0 to 18, with higher scores indicating better dietary quality for the individual (37).

### 2.5 Cardiorespiratory fitness test

This study utilized the 20-meter shuttle run test (20mSRT) to assess each student's CRF. During the 20mSRT, participants were required to run back and forth between two lines spaced 20 meters apart. The test is divided into multiple stages, each lasting approximately one minute, with an initial speed of 8.5 km/h, increasing by 0.5 km/h each minute (each minute corresponding to one stage). The test concludes when participants fail to reach the finish line in time on two consecutive occasions. The total number of completed laps was then used as the primary indicator to estimate each child's CRF level.

### 2.6 Statistical analysis methods

The Shapiro-Wilk test was used to assess the normality of continuous variables. Descriptive analysis was conducted for each variable, with normally distributed data presented as mean ± standard deviation (Mean ± SD), and independent samples t-test was used for comparisons between two groups. Non-normally distributed data were presented as median (25th percentile, 75th percentile) [Median (P25, P75)], and the Mann-Whitney U test was used for comparisons between two groups. Categorical data were reported as counts (percentages) [n (%)] and compared using the χ² test.

A generalized linear mixed model was used to analyze the associations between independent variables and various outcome indicators. To account for potential intraclass correlation among children from the same school, school was included as a random effect. Independent variables and covariates were treated as fixed effects. VFA, FINS, FPG, TG, HOMA-IR, and the 20-meter shuttle run test exhibited non-normal distribution. These variables were log-transformed to approximate normality before being included in the analysis. Model I was adjusted for sex, age, and maternal education level, while model II was further adjusted for MVPA and GDR scores.

To evaluate the mediating role of CRF in the association between screen time and CMR indicators, mediation analysis was conducted using the *mediation* package in R. Bootstrap analysis with 5,000 resamples was performed to estimate indirect effects. Statistical significance was determined through two-tailed testing, with a p-value threshold set at <0.05. All statistical analyses were conducted using R version 4.3.0 (R Core Team).

## 3 Results

### 3.1 Clinical, demographic, and screen time characteristics

A total of 1,286 participants were included in the analysis. We compared the characteristics of the included children with those of 354 non-included children (**Supplementary Table S1**), and found no significant differences between the two groups. **Table 1** presents the clinical and demographic characteristics according to the different levels of screen time. In this study, boys accounted for 52.6% and girls for 47.4%, with a mean age of 8.48 ± 0.29 years. There was no significant difference in sex distribution between the two screen-time groups (P = 0.368). Significant differences were observed between screen time groups in several indicators, including VFA (P = 0.035), WHR (P = 0.007), FPG (P = 0.014), TC (P = 0.001), LDL-C (P = 0.005), HDL-C (P = 0.001), and CRF (P < 0.001). The results indicate that children with screen time ≤2 hours per day exhibited lower levels of visceral fat, better cardiorespiratory fitness, and more favorable blood glucose and lipid profiles compared to those with screen time >2 hours per day.

**Table 1:** Basic characteristic by quantiles of screen time.

Variables	≤ 2 hours	> 2 hours	Overall	*P*
	(n=1124)	(n=162)	(n=1286)	
**Sex,n (%)**				0.368
Boys	585 (52.0)	91 (56.2)	676 (52.6)	
Girls	539 (48.0)	71 (43.8)	610 (47.4)	
**Age,years**	8.48 (0.29)	8.46 (0.30)	8.48 (0.29)	0.392
Anthropometric variables				
Height,cm	132 (5.6)	132 (5.9)	132 (5.6)	0.848
Weight,kg	29.0 (6.1)	29.8 (6.4)	29.1 (6.1)	0.162
WC,cm	57.1 (6.8)	58.1 (7.2)	57.3 (6.8)	0.114
Hip circumference,cm	70.2 (6.5)	70.4 (6.4)	70.3 (6.5)	0.735
WHR	0.81 (0.05)	0.82 (0.05)	0.81 (0.05)	**0.007**
WHtR	0.43 (0.04)	0.44 (0.05)	0.43 (0.04)	0.080
BFP,%	19.6 (7.7)	20.8 (8.3)	19.7 (7.8)	0.074
BMI, kg/m²	16.5 (2.6)	16.8 (2.7)	16.5 (2.6)	0.080
BMI Z score	0.12 (1.26)	0.33 (1.32)	0.14 (1.27)	0.052
VFA,cm²	22.3 [17.5, 29.7]	23.8 [18.5, 33.2]	22.4 [17.6, 30.1]	**0.035**
BFMI,kg/m²	3.40 (1.94)	3.70 (2.05)	3.43 (1.95)	0.079
SMI,kg/m²	4.16 (0.63)	4.20 (0.66)	4.17 (0.64)	0.540
FFMI,kg/m²	13.10 (0.95)	13.10 (1.01)	13.10 (0.96)	0.290
Blood pressure				
SBP,mmHg	102.0 (10.0)	101.0 (11.9)	102.0 (10.3)	0.812
DBP,mmHg	63.3 (7.6)	63.0 (7.1)	63.3 (7.6)	0.596
MAP,mmHg	76.0 (7.2)	75.7 (7.4)	76.0 (7.2)	0.639
Biochemical meassurements				
FINs,μU/mL	45.9 [32.9, 63.1]	47.2 [33.6, 66.7]	46.0 [32.9, 62.8]	0.806
FPG,mmol/L	4.89 [4.65, 5.12]	4.94 [4.71, 5.22]	4.89 [4.66, 5.14]	**0.014**
TG,mmol/L	0.70 [0.56, 0.91]	0.71 [0.56, 0.94]	0.70 [0.56, 0.91]	0.949
TC,mmol/L	4.70 (0.86)	4.48 (0.82)	4.67 (0.86)	**0.001**
LDL-C,mmol/L	2.84 (0.64)	2.69 (0.63)	2.82 (0.64)	**0.005**
HDL-C,mmol/L	1.60 (0.29)	1.53 (0.26)	1.59 (0.28)	**0.001**
HOMA-IR	1.45 [1.01, 1.99]	1.49 [1.05, 2.04]	1.45 [1.02, 2.01]	0.539
CMR score	-0.08 (2.93)	0.05 (3.09)	-0.07 (2.95)	0.599
**20mSRT**	24.0 [17.0, 38.0]	19.0 [15.0, 26.0]	23.0 [17.0, 36.0]	**<0.001**

WC, waist circumference; WHR, waist-to-hip ratio; WHtR, waist-to-height ratio; BFP, body fat percentage; BMI, body mass index; VFA, visceral fat area; BFMI, body fat mass index; SMI, skeletal muscle mass index; FFMI, fat-free mass index; SBP, systolic blood pressure; DBP, diastolic blood pressure; MBP, mean blood pressure; FINs, fasting insulin; FPG, fasting plasma glucose; TG, triglycerides; TC, total cholesterol; LDL-C, low-density lipoprotein cholesterol; HDL-C, high-density lipoprotein cholesterol; HOMA-IR, homeostatic model assessment for insulin resistance; CMR: cardiometabolic risk; 20mSRT: 20 – meter shuttle run test. Bold values indicate statistical significance.

### 3.2 The relationship between screen time duration and metabolic indicators of obesity

We assessed the relationship between screen time (hours per day) and metabolic indicators of obesity using two models (**Table 2**). In Model 1, higher screen time was positively associated with BMI (β = 0.131, P = 0.017), BMI Z-score (β = 0.064, P = 0.018), VFA (β = 0.031, P = 0.003), BFMI (β = 0.121, P = 0.005), BFP (β = 0.509, P = 0.003), WC (β = 0.322, P = 0.028), WHtR (β = 0.002, P = 0.039), SBP (β = 0.447, P = 0.033), and CMRs (β = 0.145, P = 0.025). Conversely, screen time was negatively associated with HDL-C (β = -0.012, P = 0.050) and CRF (β = -0.051, P < 0.001). In Model 2, after further adjusted for MVPA and GDR score, higher screen time remained positively associated with VFA (β = 0.029, P = 0.009), BFMI (β = 0.109, P = 0.017), BFP (β = 0.469, P = 0.010), while it was negatively associated with HDL-C (β = -0.014, P = 0.038), and CRF (β = -0.048, P < 0.001).

**Table 2:** Associations between screen time duration and metabolic indicators of obesity.

Variables	Model 1		Model 2	
	β (95% CI)	*p*	β (95% CI)	*P*
BMI	0.131 (0.024, 0.239)	**0.017**	0.109 (-0.006, 0.224)	0.064
BMI Z score	0.064 (0.011, 0.117)	**0.018**	0.052 (-0.004, 0.109)	0.071
VFA	0.031 (0.009, 0.051)	**0.003**	0.029 (0.006, 0.05)	**0.009**
BFMI	0.121 (0.035, 0.203)	**0.005**	0.109 (0.017, 0.197)	**0.017**
SMI	0.014 (-0.012, 0.039)	0.294	0.004 (-0.023, 0.031)	0.782
FFMI	0.019 (-0.020, 0.058)	0.353	0.007 (-0.034, 0.048)	0.740
BFP	0.509 (0.167, 0.84)	**0.003**	0.469 (0.106, 0.821)	**0.010**
WC	0.322 (0.034, 0.606)	**0.028**	0.278 (-0.027, 0.579)	0.073
WHR	0.001 (-0.001, 0.003)	0.570	0.001 (-0.001, 0.003)	0.500
WHtR	0.002 (0.0001, 0.004)	**0.039**	0.002 (0, 0.004)	0.072
SBP	0.447 (0.036, 0.858)	**0.033**	0.418 (-0.019, 0.855)	0.062
DBP	-0.029 (-0.35, 0.299)	0.861	-0.064 (-0.405, 0.283)	0.713
FPG	0.004 (-0.01, 0.018)	0.583	0.004 (-0.011, 0.019)	0.586
FINS	0.014 (-0.008, 0.036)	0.204	0.011 (-0.011, 0.034)	0.331
HOMA-IR	0.015 (-0.008, 0.038)	0.195	0.012 (-0.012, 0.037)	0.312
TG	0.012 (-0.004, 0.027)	0.147	0.012 (-0.005, 0.029)	0.152
TC	-0.023 (-0.059, 0.013)	0.220	-0.031 (-0.07, 0.007)	0.116
HDL-C	-0.012 (-0.024, 0)	**0.050**	-0.014 (-0.027, -0.001)	**0.038**
LDL-C	-0.009 (-0.036, 0.018)	0.518	-0.013 (-0.041, 0.015)	0.383
CMR score	0.145 (0.017, 0.271)	**0.025**	0.128 (-0.009, 0.262)	0.065
20mSRT	-0.051 (-0.072, -0.031)	**< 0.001**	-0.048 (-0.07, -0.026)	**< 0.001**

Model 1: adjusted for sex, age, and maternal education level

Model 2: additionally adjusted for MVPA and GDR score on the basis of model 1

WC, waist circumference; WHR, waist-to-hip ratio; WHtR, waist-to-height ratio; BFP, body fat percentage; BMI, body mass index; VFA, visceral fat area; BFMI, body fat mass index; SMI, skeletal muscle mass index; FFMI, fat-free mass index; SBP, systolic blood pressure; DBP, diastolic blood pressure; FINs, fasting insulin; FPG, fasting plasma glucose; TG, triglycerides; TC, total cholesterol; LDL-C, low-density lipoprotein cholesterol; HDL-C, high-density lipoprotein cholesterol; HOMA-IR, homeostatic model assessment for insulin resistance; CMR, cardiometabolic risk; 20mSRT, 20 – meter shuttle run test. Bold values indicate statistical significance.

### 3.3 The relationship between different levels of screen time and metabolic indicators of obesity

We categorized participants into two groups based on daily screen time: ≤ 2 hours/day and > 2 hours/day. The relationship between different levels of screen time and metabolic indicators of obesity were also examined (**Table 3**). In Model 1, screen time > 2 hours per day was positively associated with VFA (β = 0.089, P = 0.029), BFMI (β = 0.336, P = 0.047), and BFP (β = 1.611, P = 0.017), while it was negatively associated with CRF (β = -0.149, P < 0.001). In Model 2, after adjusting for covariates, screen time > 2 hours per day remained significantly associated with VFA (β = 0.088, P = 0.038), BFP (β = 1.516, P = 0.029), and CRF (β = -0.145, P = 0.001).

**Table 3:** Associations between different levels of screen time and metabolic indicators of obesity.

Variables	Model 1		Model 2	
	β (95% CI)	*p*	β (95% CI)	*p*
VFA	0.089(0.006, 0.168)	**0.029**	0.088(0.002, 0.169)	**0.038**
BFMI	0.336(-0.003, 0.661)	**0.047**	0.302(-0.047, 0.636)	0.082
BFP	1.611(0.267, 2.914)	**0.017**	1.516(0.135, 2.853)	**0.029**
HDL-C	-0.047(-0.095, 0)	0.054	-0.049(-0.098, -0.001)	**0.049**
20mSRT	-0.149(-0.231, -0.068)	**<0.001**	-0.145(-0.228, -0.063)	**0.001**

Model 1: adjusted for sex, age, and maternal education level

Model 2: additionally adjusted for MVPA and GDR score on the basis of model 1

VFA, visceral fat area; BFMI, body fat mass index; BFP, body fat percentage; HDL-C, high-density lipoprotein cholesterol; 20mSRT, 20 - meter shuttle run test. Bold values indicate statistical significance.

### 3.4 The mediating effect of CRF on the association between screen time and metabolic indicators of obesity

**Figure 2:**
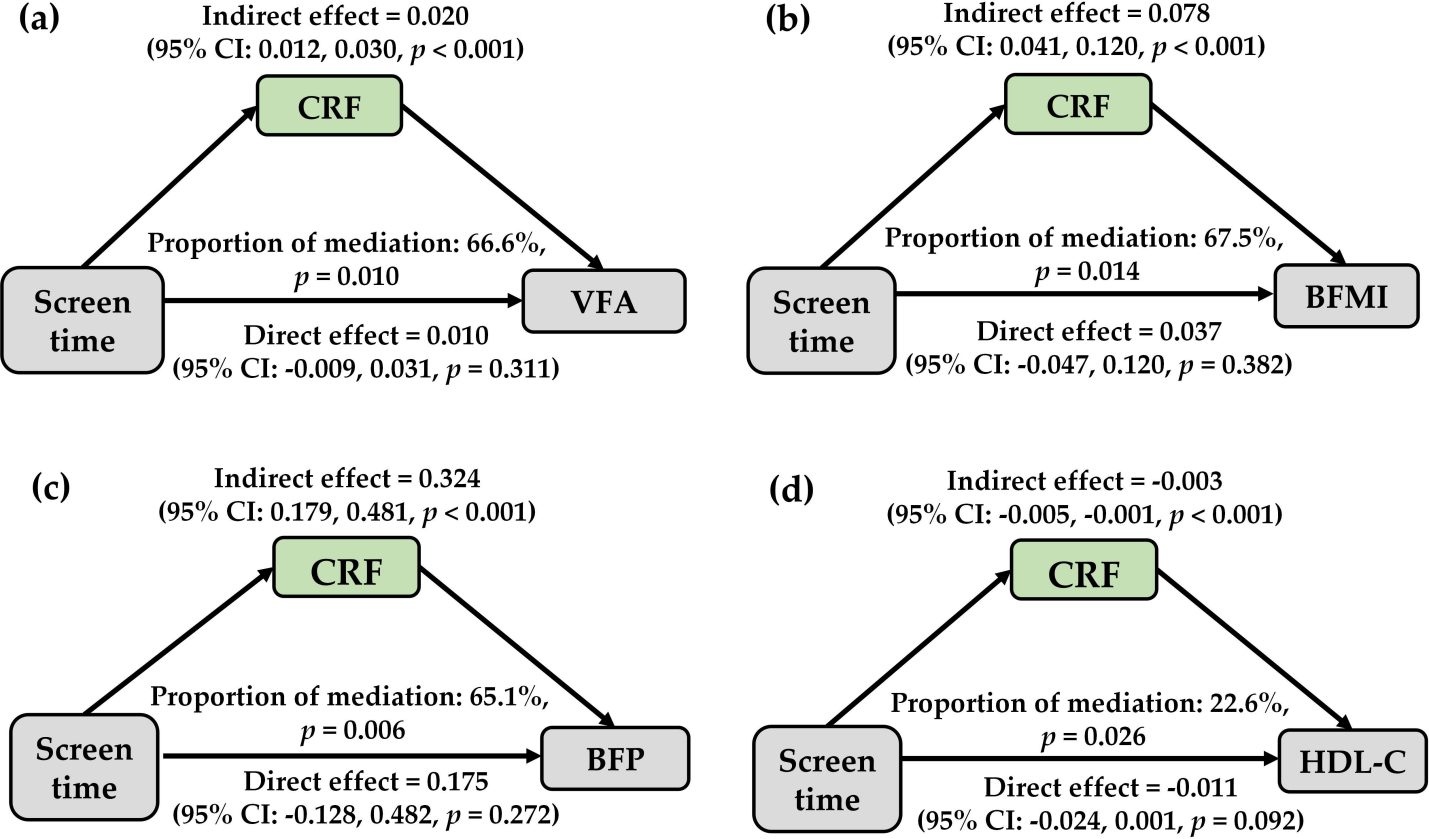
The mediating role of cardiorespiratory fitness in associations between screen time and obesity metabolic risk. **(a)** The mediation between screen time and VFA; **(b)** The mediation between screen time and BFMI; **(c)** The mediation between screen time and BFP; **(d)** The mediation between screen time and HDL-C. BFP, body fat percentage; BFMI, body fat mass index; CRF, cardiorespiratory fitness; HDL-C, high-density lipoprotein cholesterol; VFA, visceral fat area.

We investigated the mediating effect of CRF on the relationship between screen time and metabolic indicators of obesity (**Figure 2**). The results indicated that CRF mediated 66.6% (P = 0.010) of the association between screen time and VFA, 65.1% (P = 0.006) of the association with BFP, and 22.6% (P = 0.026) of the association with HDL-C. Furthermore, we found that the association between screen time and BFMI exhibited the highest mediating proportion of 67.5% (P = 0.014).

## 4 Discussion

This study examined the associations between children’s screen time, obesity-related measures, and metabolic indicators, and evaluated the mediating role of CRF. Longer screen time was positively associated with BMI, VFA, and blood glucose and lipid levels. CRF significantly mediated these associations, suggesting that higher CRF may attenuate the adverse metabolic profiles linked to excessive screen time in children.

There is a significant positive correlation between average daily screen time and obesity, with screen time identified as an important risk factor for childhood obesity and metabolic diseases, a conclusion consistent with other studies (15, 38). Our findings also support current guidelines recommending that children's screen time should be limited to less than 2 hours per day. In our study, we observed a notable increase in obesity risk among children when their cumulative screen time reached or exceeded 2 hours per day. Research indicates that children consume a substantial portion of their daily required calories while engaging with screen media (39). The influence of screen media may prolong eating duration or distract children from their feelings of fullness (40). Furthermore, while using screen devices, children are more likely to be exposed to advertisements for unhealthy foods, which can further affect their dietary preferences and choices (41). Sleep deprivation may also represent a potential link between screen media use and obesity (42). The use of blue light-emitting devices can delay sleep onset and affect sleep quality, thereby indirectly contributing to weight gain (43, 44). Future research should further investigate the underlying mechanisms connecting screen time to childhood obesity risk to develop more effective intervention strategies.

This study found that children with prolonged screen time exhibited a significant increase in body fat percentage and visceral fat area. Abnormal fat accumulation, particularly the accumulation of visceral fat, is a major contributor to obesity-related health issues. In children, similar to adults, the distribution of abdominal fat—especially the deposition of visceral or intra-abdominal fat—has a closer correlation with various cardiovascular disease and diabetes risk factors, while the impact of total body fat is relatively minor (44, 45). Visceral fat is highly metabolically active, continuously releasing free fatty acids and adipocyte-derived factors into the portal circulation. These factors can promote hyperinsulinemia, dyslipidemia, and atherosclerosis, potentially leading to increased blood pressure (46). Although cardiovascular events predominantly occur in adults, the pathological changes associated with these events often originate from early cardiovascular and metabolic abnormalities in childhood (47). Therefore, early identification and intervention of obesity issues in children and adolescents are crucial for preventing and delaying the onset of cardiovascular metabolic diseases. Visceral adipose tissue (VAT) is considered a more effective predictor for identifying unhealthy metabolic phenotypes in children and adolescents compared to traditional obesity metrics such as BMI, waist WC, and WHtR (48). Research indicates that the threshold for VFA can effectively identify cardiometabolic risk in children and adolescents, although this predictive ability varies between boys and girls. Therefore, VAT holds significant clinical relevance in predicting cardiovascular metabolic risk in children (49).

CRF is an objective health indicator that provides an accurate measure of cardiovascular health compared to self-reported physical activity levels. Research has confirmed that CRF significantly influences the relationship between screen time and obesity, as well as metabolic risk factors, particularly regarding VFA, BFP, and lipid levels. The physiological explanations for these findings are complex and may involve multiple causal pathways. For instance, higher cardiorespiratory endurance is often associated with a higher basal metabolic rate, meaning the body can expend more energy at rest (50). Simultaneously, chronic low-grade inflammation, oxidative stress, and mitochondrial dysfunction are interconnected with obesity (51, 52). It is noteworthy that these adverse physiological states can be partially improved or reversed through adequate physical activity and exercise, as well as enhancements in CRF. By promoting physical activity levels among children and adolescents and improving their cardiorespiratory endurance, it is possible to not only reduce obesity risk but also improve obesity-related metabolic health indicators.

Despite the significant strengths of this study, including a large sample size and the use of validated tools for comprehensive metabolic measurements of obesity, several limitations remain. First, as with all cross-sectional studies, we cannot infer the direction of causality between screen time and obesity. Second, screen time data were primarily collected through self-reports, which may introduce recall bias, as children are likely to underestimate their time spent on various screens. Lastly, our study population consisted of third-grade Chinese students, which may limit the generalizability of the results to different age groups and ethnicities.

In conclusion, this study provides evidence that increased screen time is associated with a higher risk of obesity and metabolic health in Chinese children, with CRF playing a significant mediating role. These findings support the development of comprehensive health promotion strategies that integrate screen time reduction and encouragement of physical activity to mitigate childhood obesity and other cardiovascular metabolic risk factors. Future research should further investigate the mechanisms underlying these relationships, particularly how enhancing cardiorespiratory fitness can alleviate obesity risk associated with excessive screen time, and consider its long-term implications for adult health.

## Data Availability

The original contributions presented in the study are included in the article/**Supplementary Material**. Further inquiries can be directed to the corresponding authors.
